# Galvanic Vestibular Stimulation Improves Spatial Cognition After Unilateral Labyrinthectomy in Mice

**DOI:** 10.3389/fneur.2021.716795

**Published:** 2021-07-29

**Authors:** Thanh Tin Nguyen, Gi-Sung Nam, Jin-Ju Kang, Gyu Cheol Han, Ji-Soo Kim, Marianne Dieterich, Sun-Young Oh

**Affiliations:** ^1^Jeonbuk National University College of Medicine, Jeonju, South Korea; ^2^Department of Neurology, Jeonbuk National University Hospital & School of Medicine, Jeonju, South Korea; ^3^Department of Pharmacology, Hue University of Medicine and Pharmacy, Hue University, Hue, Vietnam; ^4^Department of Otorhinolaryngology-Head and Neck Surgery, Chosun University College of Medicine, Kwangju, South Korea; ^5^Research Institute of Clinical Medicine of Jeonbuk National University-Jeonbuk National University Hospital, Jeonju, South Korea; ^6^Department of Otolaryngology-Head and Neck Surgery, Graduate School of Medicine, Gachon University of Medicine and Science, Incheon, South Korea; ^7^Department of Neurology, Seoul National University Hospital & School of Medicine, Seoul, South Korea; ^8^Department of Neurology, University Hospital, Ludwig-Maximilians-Universität, Munich, Germany; ^9^German Center for Vertigo and Balance Disorders-IFB, University Hospital, Ludwig-Maximilians-Universität, Munich, Germany; ^10^Munich Cluster for Systems Neurology (SyNergy), Munich, Germany

**Keywords:** vestibular, unilateral labyrinthectomy, higher vestibular cognition, galvanic vestibular stimulation, spatial navigation

## Abstract

**Objectives:** To investigate the deficits of spatial memory and navigation from unilateral vestibular deafferentation (UVD) and to determine the efficacy of galvanic vestibular stimulation (GVS) for recovery from these deficits using a mouse model of unilateral labyrinthectomy (UL).

**Methods:** Thirty-six male C57BL/6 mice were allocated into three groups that comprise a control group and two experimental groups, UVD with (GVS group) and without GVS intervention (non-GVS group). In the experimental groups, we assessed the locomotor and cognitive behavioral function before (baseline) and 3, 7, and 14 days after surgical UL, using the open field (OF), Y maze, and Morris water maze (MWM) tests. In the GVS group, the stimulations were applied for 30 min daily from postoperative day (POD) 0–4 *via* the electrodes inserted subcutaneously close to both bony labyrinths.

**Results:** Locomotion and spatial cognition were significantly impaired in the mice with UVD non-GVS group compared to the control group. GVS significantly accelerated recovery of locomotion compared to the control and non-GVS groups on PODs 3 (*p* < 0.001) and 7 (*p* < 0.05, Kruskal–Wallis and Mann–Whitney *U* tests) in the OF and Y maze tests. The mice in the GVS group were better in spatial working memory assessed with spontaneous alternation performance and spatial reference memory assessed with place recognition during the Y maze test than those in the non-GVS group on POD 3 (*p* < 0.001). In addition, the recovery of long-term spatial navigation deficits during the MWM, as indicated by the escape latency and the probe trial, was significantly better in the GVS group than in the non-GVS group 2 weeks after UVD (*p* < 0.01).

**Conclusions:** UVD impairs spatial memory, navigation, and motor coordination. GVS accelerated recoveries in short- and long-term spatial memory and navigation, as well as locomotor function in mice with UVD, and may be applied to the patients with acute unilateral vestibular failure.

## Introduction

Vestibular afferents are sensitive to motion accelerations during either head translations or rotations in space, and provide continuous information to explore and understand the enormous range of physical motions experienced in daily life (1, 2). The vestibular system functions as an inertial sensor to generate egocentric representations of space that are important for spatial perception and memory, and makes a critical contribution to spatial navigation (2–4). While other sensory systems are linearly organized, i.e., projections from the peripheral organ primarily go through a modality-specific thalamic nucleus and then to their respective cortical or subcortical areas, the vestibular information in the central nervous system (CNS) rapidly becomes multisensory, convergent, and multimodal (1, 2, 5, 6). As the vestibular projections are extensively distributed to subcortical, cortical, and cerebellar regions, (3, 5, 7) the vestibular system involves a broad variety of brain functions from automatic reflexes [vestibulo-ocular reflex (VOR)] and motor coordination to higher cognitive processes, such as spatial attention, (8) navigation, (9, 10) spatial memory, (3, 11, 12) and bodily self-consciousness (12). The navigation system is based on the information on changes in head position and direction in space and is computed during body movements (13).

Vestibular information is projected to the hippocampus *via* several long-latency and polysynaptic pathways, (4, 14–18) as the hippocampal formation is activated by vestibular stimulation (19, 20). Recent research have demonstrated that information is transmitted from the peripheral vestibular organs to the hippocampus *via* four major pathways including the thalamocortical pathway, theta-generating pathway, cerebellocortical pathway, and head direction pathway (18, 21). Inactivating the vestibular system results in the disruption of location-specific firing in hippocampal place cells, (17) which impairs the performance of animals in learning and memory tasks (22). With regard to vestibular projections to the hippocampus and cortex, more studies have recently focused on elucidating deficits in spatial navigation and memory tasks related to vestibular disorders (9). A growing number of studies have demonstrated a link between spatial cognition and vestibular impairment, especially in bilateral vestibular loss, which causes prominent and long-lasting spatial cognitive deficits (23–26). However, whether unilateral vestibular deafferentation (UVD) also causes higher vestibular cognitive deficits is a matter of debate because deficits can be hidden by a potential decline in cognitive performance with increasing age (27–32). However, neurophysiological and behavioral studies revealed that unilateral vestibular deafferented animals showed dysfunction in spatial memory and navigation (33).

Galvanic vestibular stimulation (GVS) has been used for over 100 years to investigate the role of vestibular signals in gaze, posture, locomotor control, and spatial perception under pathophysiological conditions (29, 34, 35). Weak GVS current likely operates by modulating vestibular afferents characterized by the regularity of firing rate (36–38) rather than inducing membrane depolarization of vestibular sensory organs (34, 39, 40). Recently, several studies have investigated the beneficial effects of GVS on cognitive and memory processes. In addition to the long-term efficacy of improved stroke-induced deficits in patients with spatial hemi-neglect, (41) GVS intervention also resulted in enhanced spatial memory in a rat cognitive impairment model (42).

The present study in mice was designed to investigate the deficits of spatial memory and navigation from UVD, and to determine the efficacy of GVS for recovery from these deficits using a mouse model of unilateral labyrinthectomy (UL).

## Methods

### Animals

Thirty-six male C57BL/6 mice aged 9 weeks and weighing 20–25 g (Animal Technology, Koatech, Kyonggi-Do, Korea) were randomly assigned to three experimental groups: UL with GVS intervention (GVS group, *n* = 12), UL without GVS intervention (non-GVS group, *n* = 12), and the control group (*n* = 12). Every effort was made to minimize both the number and the suffering of mice used in the experiment. Mice were acclimatized to laboratory conditions for 1 week before the experiment started and then housed separately and kept in a controlled temperature and humidity room with free access to food and water.

Both GVS and non-GVS groups underwent right-sided UL, and mice from the control group underwent sham surgery to expose the semicircular canal (SCC) without labyrinthectomy. We used surgical labyrinthectomy, which is relatively simple, reliable, and induces vestibular symptoms immediately after surgery, and has a faster recovery than vestibular neurectomy and chemical labyrinthectomy (43–45). UL was carried out according to a surgery protocol as described previously (44, 46–48). A 10-mm-long skin incision was made 5 mm behind the right auricular sulcus to expose the bony labyrinth, and the muscle and soft tissues covering the temporal bone were dissected (44, 46–48). After approaching the horizontal and posterior SCC, a small hole was made in the posterior SCC with a diamond otologic drill (0.5 mm in diameter) for perilymph leakage. Gentle suction was used to aspirate perilymph fluid for 3 min, and then the hole was filled with collagen (Helitene, Intergra Life Sciences Co., New Jersy, USA) to prevent further leakage. All treated mice were anesthetized by continuous inhalation of isoflurane gas (Ifran, O_2_ 5 L/min, 2.0, Hana Pharm Co. Ltd., Kyonggi-Do, Korea) during surgery as well as in preparation for GVS application.

The animal procedures included in this study were consistent with the Assessment and Accreditation of Laboratory Animal Care International and have been reviewed and approved by the Animal Care Committee of the Gachon University of Medicine and Science (IRB MRI2019-0008).

### Study Design and GVS Application

**Figure 1:**
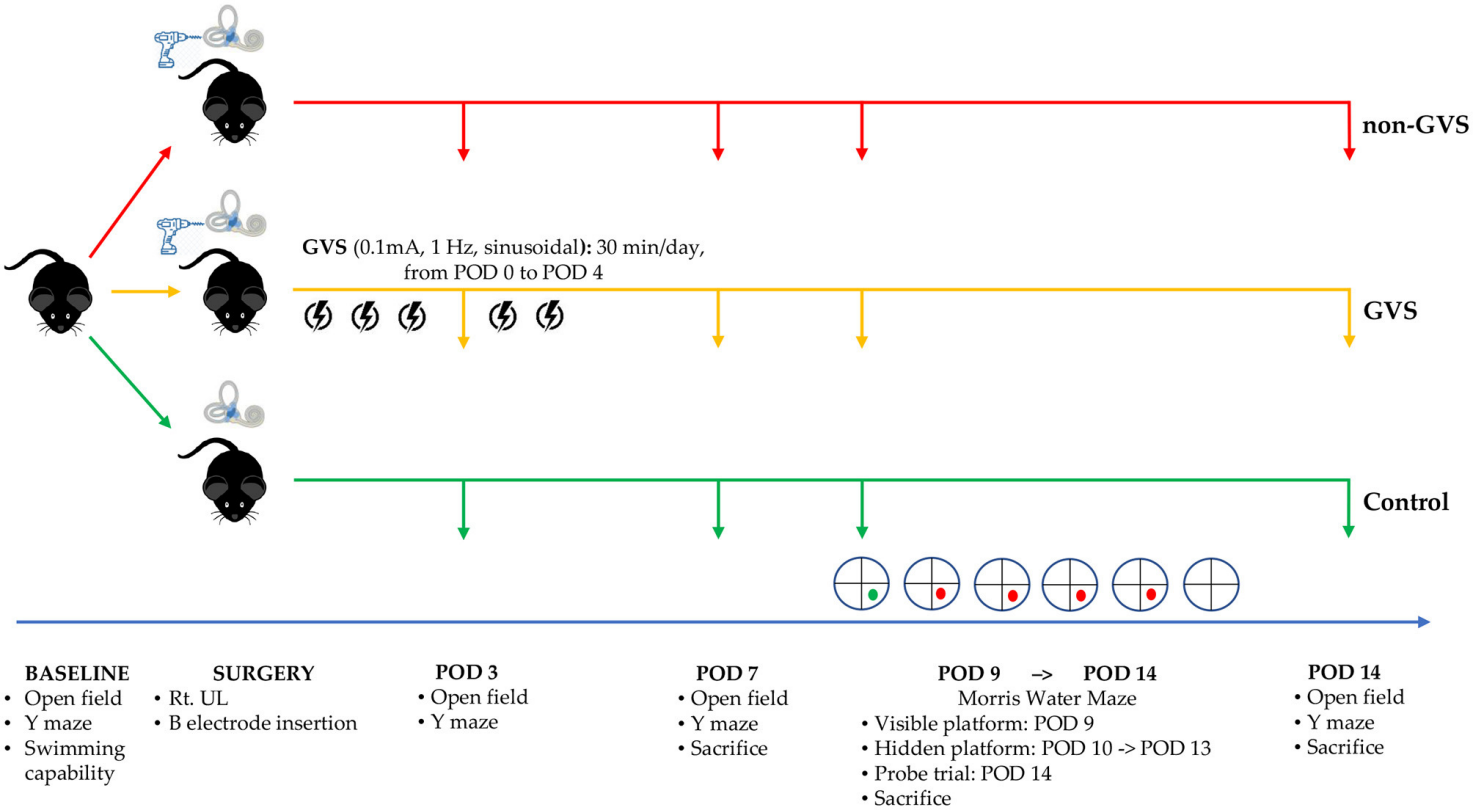
A schematic representation of the experimental design and time schedules for the application of GVS. GVS, galvanic vestibular stimulation; POD, postoperative day.

We evaluated baseline levels of swimming capacity and open field (OF) and Y maze tests before labyrinthectomy. The mice that could swim were then randomly assigned to three groups: control, non-GVS, and GVS groups. OF and Y maze behavioral tests were also used to measure locomotor activities and spatial recognition in each group on postoperative days (PODs) 3, 7, and 14 (Figure 1). GVS was delivered to the mice in the GVS group for 5 days with 30-min sessions each day from POD 0 within 5 h after UL under head-restrained awake state to POD 4. The Morris water maze (MWM) training session was started from POD 9 and continued for 5 consecutive days and the probe trial was done at POD 14 (Figure 1). To minimize the time-of-day impact on the locomotor and exploratory behavior of mice, (49) our behavioral assessments were carried out on at a certain time between 11:00 a.m. and 3:00 p.m.

For the application of GVS, we inserted electrodes made of a metal bolt cap (1.26 mm in diameter) into circular plastic buttons (5 mm in diameter) and then each electrode was attached to one uninsulated tail of a 3-cm-long wire (30 gauge) passing through the skin, which was connected to the direct current (DC) shifted galvanic stimulator (A-M Systems Model 2200 Analog Stimulus Isolator) *via* alligator connectors. After the implantation of these electrodes near bony labyrinths, the wound was closed with a 5.0 Vicryl suture to support the healing process. The sinusoidal current was generated by a computer-controlled stimulator with the cathode (excitatory) in the right (lesioned) side and the anode (inhibitory) (50) in the left (intact) side of the mice. During the pilot experiment, we determined the GVS threshold before the intervention session by delivering a sinusoidal GVS current at 1 Hz while progressively increasing intensity from zero. The GVS threshold, which exhibits a vestibular-specific effect, was the lowest level that evoked a clearly repeatable vertical-torsional nystagmus without any other muscle activity (29, 39, 51). As a result, we used a subthreshold, sinusoidal GVS current of 0.1 mA and 1 Hz for intervention in the GVS group for 5 days with 30-min sessions each day. The mice in the control and non-GVS groups were also restrained by the same procedure as in the GVS group but without current.

### OF Task

**Figure 2:**
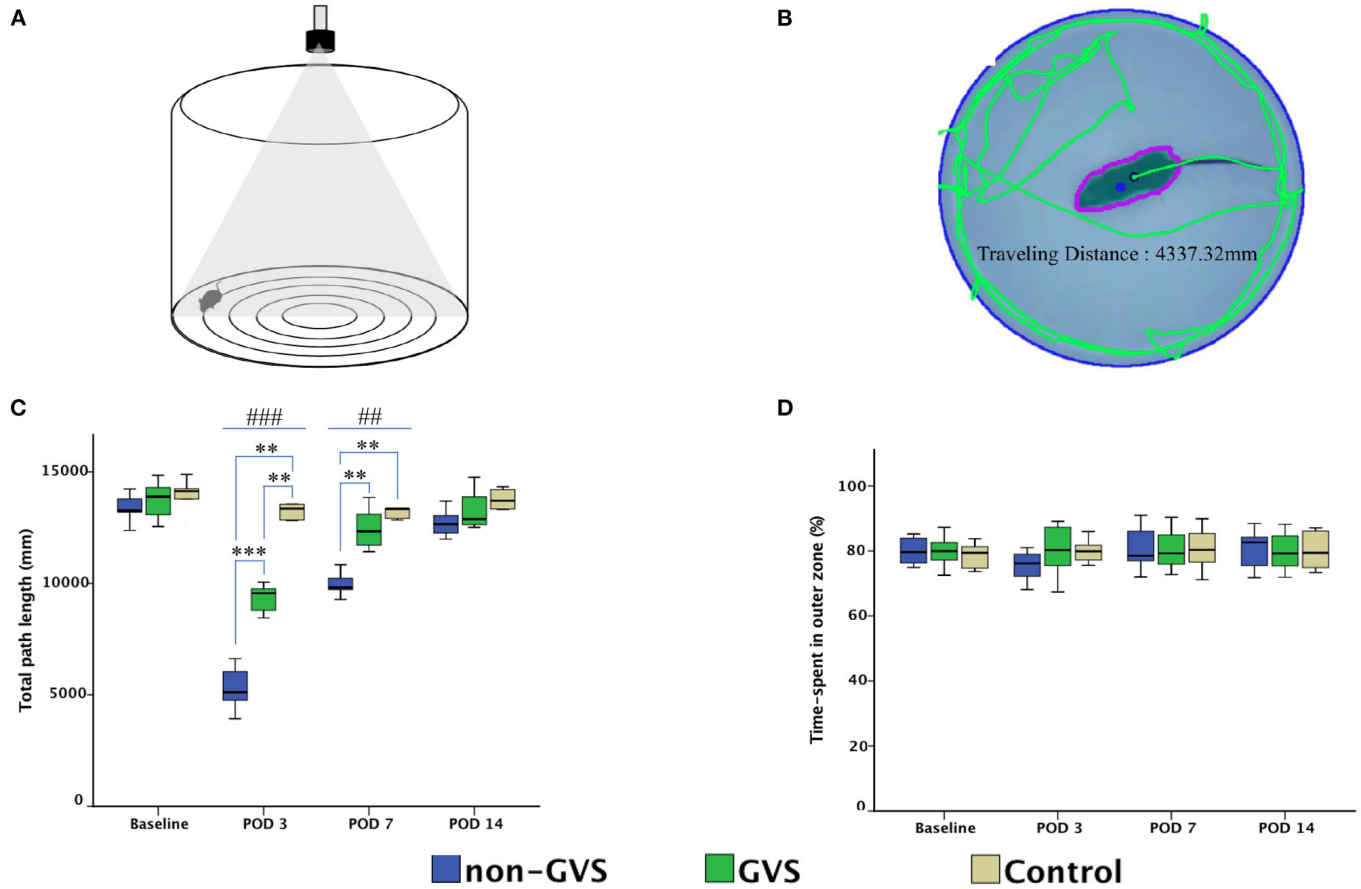
Evaluation of the locomotor activities of mice through an open field task. The open field apparatus with an overhead camera and lighting support system **(A)**. The recorded images were processed with the digital video-based tracking system using an image subtraction technique: the green lines indicate the total path length **(B)**. GVS improved the total path length during the acute phase and there are significant differences between the three groups at post-operative day (POD) 3 (χ^2^ = 17.46, *p* < 0.001, Kruskal–Wallis test) and POD 7 (χ^2^ = 14.43, *p* < 0.01, Kruskal–Wallis test) **(C)**. The percentage of time spent in the outer zone, which is an indicator of the anxiety assessment, was not different between the three groups **(D)**. *, significantly different between two groups; #, significantly different between three groups; **, ## indicate *p* < 0.01; ***, ### indicate *p* < 0.001.

Mice were tested for 2 min in OF apparatus comprising a circular arena of a white plastic cylinder with 37 cm in diameter and 53 cm in height, which was illuminated with red light from the top at the center of the apparatus (Figure 2A) (52, 53). To start each test trial, the mice were individually introduced to the center and tracked by an overhead camera HD 1080p C920 (Logitech, Switzerland) with a sampling rate of 30 frames/s. The locomotor activities of the mice were assessed by variables of the total path length for the whole device ground (mm). The ground was divided into the inner (central) and outer (peripheral) zones, and the percentage of time spent in the outer zone was used as an indicator for anxiety (52, 53). The recorded images were processed with a customized analysis package (Figure 2B) (52, 53).

### Y Maze

**Figure 3:**
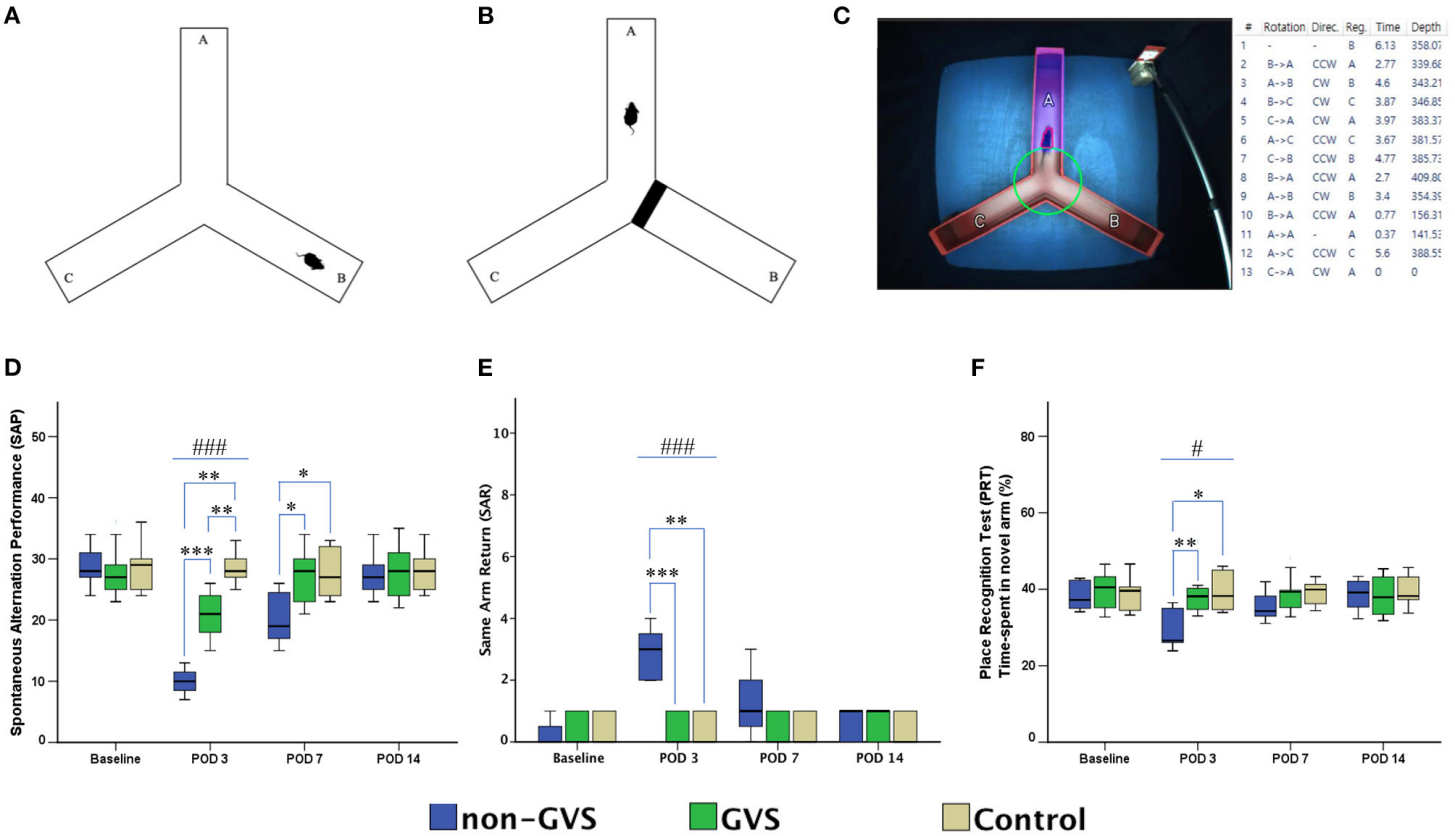
Evaluation of locomotor activities and spatial navigation through the Y maze test. The mice move freely within three arms **(A)**. Mice were trained with a block in the B arm for 3 min, then the block was removed, and the mouse activity for exploring the B arm was assessed, i.e., the place recognition test **(B)**. Mice activities in the three groups of non-GVS, GVS, and control groups were tracked and computed by analysis software in 6 min at four time points: baseline, and PODs 3, 7, and 14 **(C)**. There was a significant difference between the groups in the spontaneous alternation performance (SAP) at PODs 3 (χ ^2^ = 17.11, *p* < 0.001, Kruskal–Wallis test). This decline in the non-GVS group continued until POD 7 as compared to the GVS group (*Z* = −2.12, *p* < 0.05) and control group (*Z* = −1.95, *p* < 0.05) **(D)**. There was also a significant difference between the groups in the same arm return (SAR) at POD 3 (χ ^2^ = 15.23, *p* < 0.001, Kruskal–Wallis test) **(E)**. The place recognition test (PRT) indicates spatial reference memory and it shows a significant difference between the groups at POD 3 (χ^2^ = 7.63, *p* < 0.05, Kruskal–Wallis test) **(F)**. Values of significant difference were calculated by using the Kruskal–Wallis test for between groups and the Mann–Whitney *U* tests for pairwise comparisons. *, significantly different between two groups; #, significantly different between three groups; *, # indicate *p* < 0.05; **, ## indicate *p* < 0.01; ***, ### indicate *p* < 0.001.

A Y-shaped maze with three plastic arms (named A, B, and C) 51 cm in length, 18 cm in width, and 32 cm in height walls at an angle of 120° from each other was used (Figures 3A–C). The maze was cleaned between the test runs to get rid of odors and traces that may have unexpected effects on the test outcome. Stress influences were eliminated by acclimatizing the mice for 1 h before the experiment, allowing them to familiarize themselves with the room, smells, and noise accompanied during the experiment (54, 55). Images of mice activities throughout the task were captured by an overhead camera (30 frames/s) set at the center of the maze and used for behavioral analysis (55, 56). Starting the initial session, the mouse was introduced to the center of the maze and allowed to freely explore the three arms for 6 min. The following parameters were measured: (i) the spontaneous alternation performance (SAP), which is defined as the entries into all three arms consecutively (e.g., ABC and BCA), to evaluate spatial working memory, (54, 55) and (ii) the same arm return (SAR), which is defined as visiting the same arm repeatedly (e.g., if a mouse leaves arm A and then returns to arm A, one SAR is recorded), and reflects working memory error and typically correlates with disruption in spontaneous alternation (55–57). After several minutes of relaxation, spatial reference memory assessment was evaluated by blocking and unblocking the B arm (55). When the B arm was blocked, mice could only move freely between the A and C arms for 3 min. After unblocking the B arm, mice could move in the whole three arms for 6 min. The percentage of time spent in the B arm designated as the novel arm was used for the place recognition test (PRT) reflecting spatial working and reference memory (55, 56). These values of SAP, SAR, and PRT were measured in each group at four time points: baseline and PODs 3, 7, and 14 (Figure 1).

### Morris Water Maze

**Figure 4:**
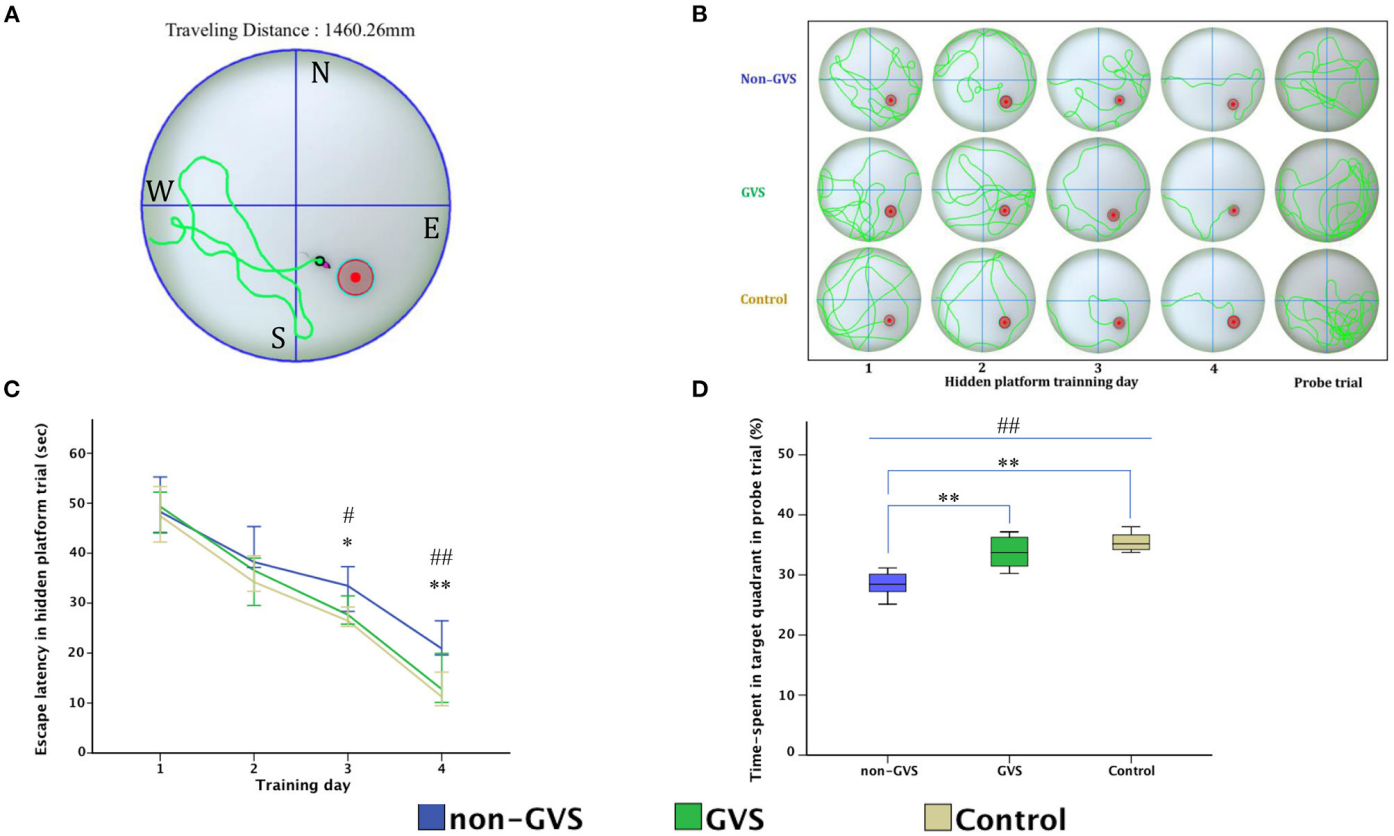
Evaluation of motor coordination and spatial navigation of mice through the Morris water maze (MWM). The analysis package divided the searching area into four quadrants, one of which contains the escape platform (red circle) **(A)**. The process of finding the escape platform from the starting point was tracked in the mice (pink) for 1 min **(A)**. Mice were trained with the visible platform at POD 8 (not depicted) and hidden platform for 4 consecutive days (POD 10–13) and no platform in the probe trial at POD 14 **(B)**. Longer values of escape latency to find the hidden platform indicate an inadequate acquisition of spatial memory and navigation, which showed differences between groups at the last two training days (χ^2^ = 6.54, *p* < 0.05 and χ^2^ = 10.52, *p* < 0.01, Kruskal–Wallis test). Non-GVS mice had a longer escape latency (33.5 s on the third day and 20.9 s on the fourth day of hidden platform trials) than those of the GVS group (27.67 s, *Z* = −2.07, *p* < 0.05 on the third day and 17.39 s, *Z* = −2.73, *p* < 0.01 on the fourth day) and the control group (26.47 s, *Z* = −2.19, *p* < 0.05 on the third day and 11.25 s, *Z* = −2.61, *p* < 0.01 on the fourth day) (Mann–Whitney *U* test) **(C)**. During the probe trial at POD 14, there was a significant decrease in the percentage of time spent in the target quadrant in the non-GVS mice (28.5% [26.2%−30.6%]) compared to the control group (35.2% [34.0%−37.3%], *Z* = −2.61, *p* < 0.01, Mann–Whitney *U* test) **(D)**. GVS intervention substantially enhanced recovery of this deficit (33.7% [30.9%−36.5%], *Z* = −2.73, *p* < 0.01, Mann–Whitney *U* test), and they were no different from the control group **(D)**. *, significantly different between two groups; #, significantly different between three groups; *, # indicate *p* < 0.05; **, ## indicate *p* < 0.01. Values of significant difference were calculated by using the Kruskal–Wallis test for between groups and the Mann–Whitney *U* tests for pairwise comparisons.

For evaluation of spatial memory and navigation, we used the MWM, which is the most basic procedure with a plastic circular water tank (175 cm diameter and 62 cm high, Jilong Frog Pool, Jilong International Co., Ltd, Hong Kong) with four starting locations of N, S, E, and W (Figure 4A) (58–60). A circular escape platform 15 cm in diameter was made of acrylic with a metal textured surface to provide traction on the top and placed in a fixed location at the center of the target quadrant (SE). It was attached to the manual laboratory scissor jack (4 × 4″ Scientific Lab Laboratory Scissor Jack, Yosoo, Shenzhen Yibai Network Technology Co. Ltd, China) to make it easier to alternate between scenarios: visible platform, hidden platform, and no platform (1.5 cm above, 1.5 cm, and 10 cm, respectively, below the surface of the water) (9). The ratio of the search area to target platform size related to task intricacy is appropriate for the 117:1 ratio of the MWM standard for mice (58). The water is made opaque by non-toxic odorless white paint, which helps to obscure the submerged platform and enables the software to locate mice by contrasting their black body with the white background of the pool. A camera of HD 1080p C920 (Logitech International SA, Lausanne, Switzerland) mounted in the center above the pool recorded the behavior of mice throughout the experiment. Mice were acclimatized to the pool and escape platform before training on POD 8. During the training session, the *visible platform trial* was conducted on POD 9, which was accompanied by the *hidden platform trial* carried out on 4 consecutive days (PODs 10–13). Each day, mice were given four trials by being lowered gently tail first into the pool facing the wall at four starting points (N, S, E, and W). Mice located the escape platform mainly depending on black triangle, red rectangle, green star, and blue circle visual cues, which were located on the surrounding walls (58, 59) rather than on the specific routes (internal self-motion cues) (58, 61). The mice were released at varying positions to exclude the turn-based trajectory to reach the platform, and they sought to use allocentric strategies to compute and remember an escape location defined by distal cues in the environment (15, 62). Each mouse was allowed 1 min to find and mount the platform. If a mouse failed to find the platform within the allotted time, it was guided to the goal and placed on the platform for 15 s. In contrast, if the goal was reached, mice remained in place for 10 s (58, 63). Mice were then removed from the pool for drying and placed in a warming cage for 5 min before returning to the home cage. Each 20-min inter-trial interval helped to eliminate the negative impact of fatigue on learning. The amount of time elapsed before the animal climbs onto the platform to escape the water (escape latency) in a hidden platform training session, measured at a fixed starting location (position W), was collected for comparison between groups (Figure 4B). In the *probe trial* (no platform), administered 24 h after the last training session, mice were released at the starting position W and swam freely for 1 min (59). The percentage of time spent in the target quadr1ant (SE quadrant) was determined to examine spatial reference memory. A visible platform test was performed 30 min after the probe trial to assess sensorimotor ability and motivation (59) that was indicated by mean swim velocity = $\frac{path\;length\;\left(mm\right)}{escape\;latency\;\left(s\right)}$.

### Statistical Analysis

All data were analyzed using SPSS Statistics version 23.0 (IBM Corp., Armonk, NY, USA). For each parameter, the normality of the distribution was assessed using Kolmogorov–Smirnov tests. Values of significant difference were calculated by using the *post-hoc* one-way analysis of variance (ANOVA) when the data follow a normal distribution; otherwise, the nonparametric Kruskal–Wallis test accompanied with Mann–Whitney *U* test or Wilcoxon signed-rank test for pairwise comparisons was used. Categorical variables were compared with chi-square test. All the tests were performed at a 0.05 level of significance.

## Results

In the acute phase after UL, signs of UVD, such as spontaneous horizontal nystagmus beating toward the contralesional side, head-tilting, falling toward the ipsilesional side, backward gait, and clockwise circling, were observed. It took about 2 days after UL for the mice to regain a stable posture and walk steadily. Considering this natural recovery course, we conducted all subsequent behavioral investigations from POD 3, free from the limitations of motor coordination problems (Figure 1).

### GVS Effect on Locomotion in UL Mice

GVS improved the total path length of OF activity during the acute phase at PODs 3 and 7. Significant differences were observed in the total path length (mm) between the three groups at PODs 3 (χ^2^ = 17.46, *p* < 0.001, Kruskal–Wallis test) and 7 (χ^2^ = 14.43, *p* < 0.01, Kruskal–Wallis test) (Figure 2C). The between-group analysis revealed that the mean total path length was decreased in the non-GVS group [5,114.7 (4,557.9–6,156.7) mm] compared to the GVS group [9,558.7 (8,760.8–9,844.6) mm] (*Z* = –3.33, *p* < 0.001, Mann–Whitney *U* test) and the control group [13,356.3 (12,837.7–14,199.5) mm] (*Z* = –2.84, *p* < 0.01, Mann–Whitney *U* test) at POD 3. This trend persisted until POD 7 when the total path length decreased in the non-GVS group [9,812.6 (9,706.3–10,540.0) mm] compared to the GVS (*Z* = −3.33, *p* < 0.001, Mann–Whitney *U* test) and control (*Z* = −2.84, *p* < 0.01, Mann–Whitney *U* test) groups.

However, the percentage of the time spent in the outer zone did not reveal differences between the three groups and the mice tended to remain in the proximity of the wall as a normal phenomenon (Figure 2D). This behavior is interpreted as an indicator of anxiety, (53, 64, 65) based on the assumption that the central area is more threatening for rodents than its periphery (44). This is supported by the increase in the center occupation following anxiolytic drug administration (66, 67). It suggests that the negative impact of anxiety on locomotor and spatial cognition in the current study was negligible.

### Spatial Cognition in UL Mice and GVS Effects

 The alternation performance and spatial recognition/attention reflected by SAP and SAR during the Y maze were disrupted in UL mice. The SAP, which is an indicator of spatial working memory as well as locomotor activity, was decreased during the acute phase at POD 3 in both labyrinthectomized groups (GVS group; *Z* = −2.87, *p* < 0.01, non-GVS groups; *Z* = −2.84, *p* < 0.01, Mann–Whitney *U* test) compared to the control group (χ^2^ = 17.11, *p* < 0.001, Kruskal–Wallis test). However, the mice with GVS intervention (GVS group) alternated between the arms of the maze more frequently, i.e., increased number of arm entries than the mice without GVS (non-GVS group) at PODs 3 (*Z* = −3.34, *p* < 0.001, Mann–Whitney *U* test) and 7 (*Z* = −2.12, *p* < 0.05, Mann–Whitney *U* test) (Figure 3D).

The SAR was scored as cumulative returns into the same arm and suggests the degree of attentional difficulties during active working memory performance. The number of SAR was significantly increased in the non-GVS group (3 [2-4] turns) compared to the GVS group (0 [0–1] turns, *Z* = −3.45, *p* < 0.001, Mann–Whitney *U* test) and the control group (0 [0–1] turns, *Z* = −2.93, *p* < 0.01, Mann–Whitney *U* test) at the acute period of POD 3 (χ^2^ = 15.23, *p* < 0.001, Kruskal–Wallis test). There was no difference between the UL mice with the GVS intervention (GVS group) and the control group (Figure 3E). The PRT, which is an indicator of spatial reference memory, was significantly different between groups at the acute phase of POD 3. The mean time spent in the novel arm was significantly increased after GVS intervention (GVS group) compared to the non-GVS group (38.1% in the GVS group vs. 26.6% in the non-GVS group, *Z* = −2.59, *p* < 0.01, Mann–Whitney *U* test) at POD 3 (Figure 3F). The improvement in visiting the novel arm in the GVS gbbroup reached the value of the control group; i.e., there was no difference between the GVS group and the control group at POD 3. However, at the subacute phase of PODs 7 and 14, there were no differences in the values of PRT between groups.

During the MWM, the escape latencies to find the hidden platform gradually decreased through the training sessions (Figures 4B,C). Longer values of escape latency to find the hidden platform indicate an inadequate acquisition of spatial memory and navigation, which showed differences between the groups on the last two training days (χ^2^ = 6.54, *p* < 0.05 and χ^2^ = 10.52, *p* < 0.01, Kruskal–Wallis test, Figure 4C). In particular, GVS intervention shortened escape latency and revealed that non-GVS mice showed longer escape latency (33.5 s on the third day and 20.9 s on the fourth day of hidden platform trials) than the GVS group (27.67 s, *Z* = −2.07, *p* < 0.05 on the third day and 17.39 s, *Z* = −2.73, *p* < 0.01 on the fourth day) and the control group (26.47 s, *Z* = −2.19, *p* < 0.05 on the third day and 11.25 s, *Z* = −2.61, *p* < 0.01 on the fourth day) (Mann–Whitney *U* test) (Figure 4C). During the probe trial at POD 14, the GVS intervention also showed significant effects with an increased percentage of time spent in the target quadrant in the GVS group compared to the non-GVS group (33.7 [30.9–36.5]% vs. 28.5 [26.2–30.6]%, *Z* = −2.73, *p* < 0.01, Mann–Whitney *U* test), which is comparable to the control group (35.2 [34.0–37.304]%, *Z* = −1.13, *p* > 0.05, Mann–Whitney *U* test) (Figure 4D). Because there were no significant differences in the mean swim velocity between groups (χ^2^ = 3.26, *p* > 0.05), these MWM learning impairments were not specific to vestibulo-motor deficits (58, 68).

## Discussion

The current study accentuates the clear effect of GVS intervention on spatial memory and navigation as well as on locomotion induced by UVD in the mouse model.

### Unilateral Vestibular Loss and Spatial Cognition

Although the vestibular system integrates the multisensory signals between the ipsilateral and contralateral side of the multi-level brain regions, the current study and other neurophysiological and behavioral studies revealed that animals who lost one-half of their vestibular afferents (UVD) showed dysfunction in spatial memory and navigation in the acute phase after UL (33). The hippocampus, along with other medial temporal lobe structures, uses information from the vestibular system to build up maps of 3D space that can be used in the development of spatial memory during learning tasks (14, 30, 69, 70). In the current experiment, short- and long-term spatial memory deficiencies in the mice with UVD were analyzed using the Y maze and MWM tasks, respectively.

The SAP during Y maze is driven by the innate curiosity of rodents to explore novel environments and requires good spatial working memory to remember the arms that have already been visited to enter a less-visited arm, resulting in increased alternation rates and reducing retention intervals (71). While performing PRT, mice should remember the relationship between distal spatial cues to the arm, which referred to the arm that had previously been blocked and not yet explored as novel, and thus visit it more frequently than the other arms (55). Both SAP and PRT have been used for measuring spatial working and reference memory, (54) especially the short-term memory component. The MWM was also designed as a method to assess hippocampal-dependent spatial navigation and reference memory, especially in place learning with extensive evidence of its validation (9). However, the MWM task was considered more specific for spatial navigation than PRT because MWM excludes the use of non-spatial or proximal cues to solve the maze, such as the odor trail interference in the Y maze (61). Furthermore, the MWM reflects the long-term memory or consolidation process in the hippocampus rather than the immediate and short-term effects of unilateral vestibular loss due to the 24-h interval between the training session and the probe trial session (72–74). It has been shown that short-term and different stages of long-term memory are not sequentially linked despite the consolidation of new memory into the long-term memory being time-dependent (72, 75).

A small-animal positron emission tomography (micro-PET) study in mice revealed significant asymmetric changes of glucose metabolism in the vestibulocerebellum, amygdala, and hippocampus during the acute phase after UL (76). In neurochemical studies in rats, UVD leads to biochemical changes such as nitric oxide (NO), which has been implicated in the mechanisms of hippocampal synaptic plasticity associated with the development of short-term spatial and non-spatial memories, (77–80) in the ipsilateral and contralateral hippocampus within several hours after UL (78, 80). Another experiment showed that UL caused time-dependent changes in nitric oxide synthase (NOS) activity in the hippocampal information (78, 80). After UVD, a significant increase in NOS activity occurred in the ipsilateral dentate gyrus within several hours and persisted for several days (81). Thereafter, there was a long-term decrease in neuronal NOS expression in the ipsilateral dentate gyrus (78) and also in the N-methyl-D-aspartate (NMDA) receptor subunit expression in the ipsilateral hippocampal CA2/3 region (82) at 2 weeks after unilateral vestibular lesion. Other studies in rodents have shown that UL results in bilateral changes in electrical excitability of the hippocampal CA1 region, which is a critical structure for spatial navigation and memory, (79, 83) and revealed deficits in navigation ability (70). Cellular mechanisms for learning and memory storage in the hippocampus can be explained with long-term potentiation (LTP), a long-lasting enhancement of excitatory postsynaptic field potentials, which is easily induced by high-frequency electrical stimulation in the CA1 area (84). A substantial reduction in LTP induction in bilateral CA1 areas with ipsilateral predominance was observed from the day after surgical UL and persisted until 1 month after UL (83). Intriguingly, this phenomenon has been demonstrated in hippocampal slices *in vitro* post-UL but not *in vivo* following bilateral vestibular lesions in rats (85, 86). This discrepancy may be due to the type of lesion (UVD vs. BVD), the different stimulation paradigms, or *in vitro*/*in vivo* differences. Likewise, a remarkable decrease in electrical excitability in the response of bilateral CA1 neurons to stimulation of the Schaffer collateral pathway, which are axons from the CA3 region of the hippocampus to the CA1 region, lasted for 5–6 months after UL (79). These studies have shown that unilateral loss of peripheral vestibular inputs may result in profound changes in synaptic excitability of the hippocampal CA1 area during the acute stages of vestibular compensation. Therefore, it is presumed to be part of a mechanism that the hippocampus integrates allocentric and egocentric signals to create a representation of the three-dimensional spatial environment (13). On the other hand, the release of glucocorticoids during the early phase of recovery is associated with vestibular compensation following peripheral vestibular damage (87). A significant decrease in glucocorticoid receptor expression in the ipsilateral CA1 at 2 weeks after UL in rats, suggesting deficits of hippocampal function and spatial cognition following the unilateral vestibular damage, was reported (87). In experiments with MWM-trained mice, gene expressions associated with synaptic plasticity (e.g., spinophilin, activity-regulated cytoskeleton-associated protein, and neurogranin) were increased in multiple brain regions, particularly in the hippocampus (59, 88–90). Interestingly, the immediate-early gene expression such as Arc, zif268, and c-fos gene expression, (45, 91, 92) which may have a critical role in memory consolidation processes, were also altered after UL in the hippocampal neurons (93). Many converging lines of evidence provide reasonable explanations for short- and long-term spatial memory and navigation deficits after UL in rodents, as found in the current results in mice.

### GVS Effects on Locomotion and Spatial Cognition

Basically, GVS current is likely to operate by modulating the firing rate of the vestibular afferents, which is excitatory at the cathode and inhibitory at the anode, (36, 38) rather than by inducing membrane depolarization and generating series of action potentials of the vestibular sensory organ (34, 39, 40). By placing the cathode (excitatory) on the right (lesioned) side and the anode (inhibitory) (50) on the left (intact) side of the mice, we aimed to rebalance the firing rate by attenuating the intact side and facilitating the lesioned side. In a preceding GVS intervention study using the rotary test in chemical UL rats, static and dynamic vestibular compensation were accelerated in the GVS group at 2 weeks after UL (29). The authors interpreted the positive effects of GVS on the vestibulo-spinal and other non-dopaminergic pathways as a kind of neuromodulation mechanism probably resulting from the facilitatory effect on the vestibular nuclei (VN). These findings are consistent with our current study showing significant increases in the total path length of OF and spontaneous alternative behavior during Y maze tasks after GVS intervention, which implies the positive effects of GVS application on locomotor and dynamic postural control during the acute phase after UL. Neuronal sensitivity for GVS increases with discharge variability, whereby the thick fast-conducting irregularly firing afferents were more sensitive than the thin slower-conducting regularly firing vestibular afferents for both cathode and anode (34, 37, 39, 94). In contrast to regularly firing afferents, which prevail on the input of VOR neurons, the irregularly firing afferent fibers are preferentially connected with vestibulo-spinal neurons (34, 95) that underlie the postural asymmetries after UL (28, 96, 97). Therefore, GVS intervention can restore locomotor function *via* modulating type I hair cells, (37, 39) which shows an irregular phasic signal (40, 98) and may accelerate vestibulo-motor compensation after UL during the acute period.

In the current study, GVS intervention was also revealed to accelerate short- and long-term spatial working and reference memory recoveries in UL mice, which was in line with a previous rodent study on cognitive impairment induced by intraventricular administration of streptozotocin (42). A micro-PET study revealed that the excitatory GVS applied on the right side activates the left hippocampus, entorhinal cortex, and cingulate cortex (99). Another study revealed that electrical stimulation excited the medial VN and increased the firing rates of hippocampal CA1 complex spike cells corresponding to place cells (100). Similarly, GVS located at the ampulla of the semicircular canal generated the initiation of theta activity in numerous areas of hippocampal formation, (14) and it can be speculated that GVS improves neuronal activity for spatial orientation (101, 102). The hippocampal theta rhythm plays a pivotal role in spatial information processing and modulates self-movement signals (103). Additionally, an increase of c-Fos positive cells in the hippocampus, which is an indicator of neuronal activation, was documented following subsequent repetition of GVS (42, 92). Therefore, the current effect of GVS intervention on spatial memory and navigation tasks may be due in part to frequent activation of the vestibular hair cells and of neurons in the VN and also the hippocampus.

In addition to enhancing the recovery of vestibular function, GVS intervention concurrently ameliorates the function of other sensory systems such as visual and somatosensory functions, (99) which also work efficiently on spatial memory and navigation tasks (104). Another possible explanation for the benefit of GVS intervention comes from non-specific arousal, i.e., sensory stimulations such as enhancing automatic orientation by restoration of the internal representation for the egocentric frame. This seems to modulate the cortical and non-cortical areas involved in spatial cognition (105, 106). Therefore, GVS presumably enhances the function of spatial navigation through multimodal mechanisms.

The improvement of vestibular lesion-induced deficits of cognition raised the question and debate whether GVS also improves spatial memory and cognition in healthy individuals. In a study with normal rats, high-amplitude GVS showed negative effects on cell proliferation and possibly neurogenesis in the hippocampus but no significant effects on spatial memory (51). This discrepancy with our current study is very likely due to the differences between UVD and labyrinthine intact animals, since this nicely fits with data on GVS in healthy participants for a spatial orientation (random number generation) task that showed no effects on number space (107, 108). Patients with an acute unilateral peripheral vestibular deficit showed no bias in number space but a worse performance in generating sequences of random numbers during active head turns (108). The different results could further be influenced by the different GVS parameters or species in both studies. In contrast to our repetitive subthreshold GVS, Zheng and coworkers (51) used a single suprathreshold high-amplitude GVS that increased the error rates for match-to-sample tasks compared to a subthreshold GVS group, and thereby impaired hippocampal cell proliferation and neurogenesis (35, 51). So far, it is unclear which GVS parameter has a striking positive effect on spatial memory and cognition; this should be addressed in future research.

In conclusion, this is the first study on GVS intervention to investigate functional recovery for locomotion and spatial navigation in the UL mice model. There was an accelerated recovery of the spatial memory and navigation deficits during the acute phase caused by surgical UL. This finding could have important implications for the management of patients with unilateral vestibular damage who may suffer from substantial cognitive impairment. Even if UL had limited impact, since spatial cognitive deficits substantially improved within 2 weeks, the comprehensive efficacy of GVS intervention in spatial memory and navigation deficits remains to be clarified by further experiments with functional and structural imaging in patients with bilateral vestibular dysfunction who suffer from persistent deficits of spatial orientation and navigation.

## Data Availability Statement

The original contributions presented in the study are included in the article/supplementary material, further inquiries can be directed to the corresponding author/s.

## Ethics Statement

The animal procedures included in this study were consistent with the Assessment and Accreditation of Laboratory Animal Care International and have been reviewed and approved by the Animal Care Committee of the Gachon University of Medicine and Science (IRB MRI2019-0008).

## Author Contributions

S-YO and GC Han contributed on the conception or design of the work. TTN, G-SN, and J-JK conducted experiments and data collection. S-YO, J-SK, MD, and GC Han analyzed the data and interpretation. TTN and S-YO drafted the article. All authors contributed to the article and approved the submitted version.

## Conflict of Interest

The authors declare that the research was conducted in the absence of any commercial or financial relationships that could be construed as a potential conflict of interest.

## Publisher's Note

All claims expressed in this article are solely those of the authors and do not necessarily represent those of their affiliated organizations, or those of the publisher, the editors and the reviewers. Any product that may be evaluated in this article, or claim that may be made by its manufacturer, is not guaranteed or endorsed by the publisher.
